# Differences between bipolar disorder types 1 and 2 support the DSM two-syndrome concept

**DOI:** 10.1186/s40345-022-00268-2

**Published:** 2022-08-03

**Authors:** Leonardo Tondo, Alessandro Miola, Marco Pinna, Martina Contu, Ross J. Baldessarini

**Affiliations:** 1 International Consortium for Mood & Psychotic Disorders Research, McLean Hospital, Belmont, MA United States; 2 Department of Psychiatry, Harvard Medical School, Boston, MA United States; 3 Lucio Bini Mood Disorder Center, Via Cavalcanti 28, Cagliari, Italy; 4 Department of Neuroscience, Padova Neuroscience Center, University of Padova, Padua, Italy; 5 Section of Psychiatry, Department of Medical Science and Public Health, University of Cagliari, Cagliari, Italy

**Keywords:** Affective, Bipolar disorder, Comparisons, DSM-5, Types I and II

## Abstract

**Objective:**

To compare characteristics of bipolar disorder patients diagnosed as DSM-5 types I (BD-1) vs. II (BD-2).

**Methods:**

We compared descriptive, psychopathological, and treatment characteristics in a sample of 1377 consenting, closely and repeatedly evaluated adult BD patient-subjects from a specialty clinic, using bivariate methods and logistic multivariable modeling.

**Results:**

Factors found more among BD-2 > BD-1 cases included: [a] *descriptors* (more familial affective disorder, older at onset, diagnosis and first-treatment, more education, employment and higher socioeconomic status, more marriage and children, and less obesity); [b] *morbidity* (more general medical diagnoses, less drug abuse and smoking, more initial depression and less [hypo]mania or psychosis, longer episodes, higher intake depression and anxiety ratings, less mood-switching with antidepressants, less seasonal mood-change, greater %-time depressed and less [hypo]manic, fewer hospitalizations, more depression-predominant polarity, DMI > MDI course-pattern, and less violent suicidal behavior); [c] specific *item-scores* with initial HDRS_21_ (higher scores for depression, guilt, suicidality, insomnia, anxiety, agitation, gastrointestinal symptoms, hypochondriasis and weight-loss, with less psychomotor retardation, depersonalization, or paranoia); and [d] *treatment* (less use of lithium or antipsychotics, more antidepressant and benzodiazepine treatment).

**Conclusions:**

BD-2 was characterized by more prominent and longer depressions with some hypomania and mixed-features but not mania and rarely psychosis. BD-2 subjects had higher socioeconomic and functional status but also high levels of long-term morbidity and suicidal risk. Accordingly, BD-2 is dissimilar to, but not necessarily less severe than BD-1, consistent with being distinct syndromes.

## Background

The Kraepelinian proposal of a broad *manic-depressive insanity* concept was followed by more than a century of contentious efforts to subdivide it into component syndromes (Trede et al. 2005). Dunner and his colleagues proposed a second type of bipolar disorder (BD-2) in the 1970s, considered to be distinguished by lack of manic or psychotic episodes (Dunner et al. 1976). Other studies favored such differentiation based on genetic features, family history, and illness-course (DePaulo and Simpson 1987; Endicott et al. 1985), and supported a prophylactic effect of lithium in BD-2 as well as BD-1 (Peselow et al. 1982). Differentiation of BD-2 from BD-1 depends greatly on the concept of *hypomania* introduced in 1881 by Emanuel Mendel (1837–1907) and later by Carl Gustav Jung (1875–1961), which can occur in both BD-1 and BD-2, but without mania in BD-2 (Shorter 2005).

The decision to first include the diagnosis of BD-2 in DSM-IV was not easy (Frances and Jones 2012). At issue were whether: [a] to leave BD-2 as a boundary case within the category of unipolar major depressive disorder (MDD) as in DSM-III-R; [b] to add a specifier "with hypomanic episodes" to MDD, or [c] to add BD-2 as a new diagnosis within a *bipolar spectrum*. There was a need to balance risk of over-diagnosing BD-2 and prescribing possibly unneeded mood-stabilizers to MDD patients or underdiagnosing BD-2 and exposing type I BD (BD-1) patients to potentially ineffective or harmful antidepressants.

The distinction of BD-1 and BD-2 was maintained in DSM-5 as well as in ICD-11. Both systems consider BD-2 to involve recurrences of major depressive episodes with elevations of mood and activity never more severe than hypomania and rarely involving psychosis, particularly in [hypo]manic phases. However, for BD-2 DSM-5 does not recognize specifiers provided for BD-1, including polarity of a most recent episode, severity of episodes, presence of mixed/psychotic features, or level of remission. It has also become clear that mood-stabilizing treatments can be beneficial with BD-2 as well as BD-1 (Fieve et al. 1976; Kane et al. 1982), whereas antidepressants often prove to be less helpful and sometimes destabilizing (Kukopulos et al. 1980; Ghaemi 2008), and antipsychotics and other antimanic treatments usually are not necessary for hypomania in BD-2 (Baldessarini 2013; Swartz and Suppes 2019).

Despite these developments, BD-2 continues to be a relatively "difficult" syndrome to identify and distinguish clinically, particularly from MDD (Swartz and Suppes 2019; Vieta and Suppes 2008). Difficulty arises largely from the challenge of identifying hypomania retrospectively, especially as many BD-2 patients fail to recognize hypomania with their elevated mood as pathological or in need of clinical help. An emerging challenge for BD-2 is that defining optimal treatment for acute episodes and long-term prophylaxis awaits adequate research (Swartz and Suppes 2019). In addition, the status of BD-2 as distinct from BD-1 has been questioned recently. The Australian and New Zealand College of Psychiatrists recently decided not to recognize BD-2 (Malhi et al. 2020), whereas retaining BD-2 is supported by some bipolar disorder experts, notably Parker (Parker 2021) but not by Gitlin and Malhi (2020) who favor a dimensional, single-entity model of BD. This model would consider BD-2 as marked by depressions without manias, but nevertheless requiring clinical differentiation from MDD and appropriate treatment.

These uncertainties led us to carry out a systematic comparison of descriptive and clinical characteristics of a large sample of extensively and repeatedly evaluated study participants who consistently met DSM-5 diagnostic criteria for BD-1 or BD-2.

## Methods

### Subjects and clinical assessment

This study included 1377 consecutive adult patients, followed for an average of 18.5 [1–46] years at the Lucio Bini Psychiatric Center in Cagliari, Sardinia—a specialized, academic, outpatient clinic for diagnosis, treatment and research in affective disorders. Included were consenting adults who consistently met DSM-5 criteria for a primary diagnosis of BD-1 or BD-2. All participants underwent systematic initial and repeated diagnostic evaluations during follow-up by the same clinical expert (LT), based on semi-structured interviews developed at the clinic and in use since 1977. Standard clinical rating scales were used at intake and some were used repeatedly at follow-up assessments.

Clinical data acquired were recorded systematically and converted to digitized form, with diagnoses updated to meet DSM-5 criteria. Participants provided written informed consent at clinic entry for collection and analysis of their data to be presented anonymously in aggregate form for research purposes, in accordance with the requirements of Italian law.

### Factors included

We considered *demographic and general descriptive factors* including age, sex, education, marital status, employment, socioeconomic status, body-mass index (BMI); *clinical factors* that include family history of psychiatric illnesses, mood disorders or BD, and of suicidal behavior; co-occurring substance abuse (including cigarette smoking), attention deficit disorder (ADHD); anxiety disorders, or medical illnesses; presence of suicidal ideation or behavior, age at first suicide attempt, and types of suicidal acts (nonviolent, violent); and the most frequent rate of spring/summer seasonality for [hypo]mania.

For *psychometric features* at clinic intake, we included affective temperament with the 39-item Temperament Evaluation of Memphis, Pisa, San Diego scale (TEMPS-A) (Akiskal et al. 2005); depression using the 21-item Hamilton Depression Rating Scale (HDRS_21_) (Hamilton 1960) and the Montgomery-Åsberg Depression Rating Scale (MADRS) (Montgomery and Åsberg 1979); likelihood of lifetime presence of BD with the Mood Disorder Questionnaire (MDQ) (Hirschfeld et al. 2000); current [hypo]manic status with the Young Mania Rating Scale (YMRS) (Young et al. 1978), and anxiety with the Hamilton Anxiety Rating Scale (HARS) (Hamilton 1959). HDRS_21_ and YMRS were also administered at follow-up visits. Possible differences in depressive symptomatology, were assessed comparing values for each item of the HDRS_21_ in BD-1 vs. BD-2 subjects, and we considered improvement of depression ratings with initial treatment. Values > 1 for item 9 of the HDRS and 5 of the YMRS supported the specification of mixed features.

Among illness course-related variables we included: age at first symptoms, first syndromal episode, first diagnosis, and first treatment; type and duration of first lifetime episode, either depressive, psychotic, mixed, manic or hypomanic. Mixed features were diagnosed if dysphoria or agitation were present. Moreover, we assessed the course sequence (Depression-[Hypo]mania-euthymic Interval [DMI] or [Hypo]mania-Depression-Interval [MDI]), and presence of rapid cycling (≥ 4 episodes in a year); predominant polarity (≥ 50% of time ill in depression vs. [hypo]mania); and factors associated with *morbidity* by means of life charting in use at the clinic since 1977 as the observed frequency of illness-episodes (episodes/year, depressions/year, [hypo]manias/year) and the average proportion of time in [hypo]mania or depression before intake and during follow-up, as well as the presence of episodes with mixed or psychotic features, predominant (≥ 50% of follow-up time) mood, and rapid (in less than one week) switching from depression to [hypo]mania during treatment with an antidepressant.

During follow-up we compared prevalence of particular types of *treatment*, including lithium salts, antidepressants, anticonvulsants, antipsychotics, or benzodiazepines, as well as psychotherapy, given based on clinical assessments of symptomatic presentation and diagnosis.

### Statistical analysis

Data are presented as means with 95% confidence intervals (CI). Initial bivariate comparisons of characteristics of types 1 and 2 BD subjects used contingency tables (χ^2^) for categorical measures and analysis of variance (*t*-score) for continuous measures. Characteristics were tabulated as: [a] descriptive-sociodemographic, [b] morbidity, [c] HDRS item scores, and [d] treatments. The high number of subjects mean that specific tests for normality of data distribution were not required. Preliminary bivariate analyses were considered without adjustment for multiple comparisons to guide selection of factors with *p* < 0.10 for stepwise entry into multivariable logistic modeling to generate Odds Ratios (OR) and their CI to test for factors that significantly and independently differentiated BD-2 from BD-1. Factors with ≥ 10% missing values were not included. Analyses employed commercial software: *Statview.5* (SAS Institute, Cary, NC) for spreadsheets, and *Stata.13* (StataCorp, College Station, TX) for analyses.

## Results

### Descriptive comparisons

Of the 1377 participants, 47.7% were diagnosed with BD-1 and 52.3% with BD-2, respectively (ratio: 1.10); 54.3% and 59.4% of participants were women, and current age averaged 43.2 and 48.4 years (Table 1). BD-2 subjects were more likely to have psychiatric illnesses including mood disorders among first-degree relatives, but neither more BD (Table 1) nor suicidal behavior. BD-2 subjects were significantly older at clinic intake and at first reported psychiatric symptoms, first syndromal presentation, and first diagnosis and treatment, but latency between initial symptoms and first psychiatric treatment was similar with BD-1 and BD-2, averaging 6.29 years. Also similar was the overall duration of exposure to illness (Table 1). BD-2 subjects also were more likely to be educated beyond high school, employed, of medium or higher socioeconomic status (SES), married and with children, but with similar rates of marital separation (Table 1). BD-1 subjects had higher average BMI and were more likely to have a BMI ≥ 30 kg/m^2^. Assessment of temperament indicated significantly higher ratings for cyclothymic temperament among BD-2 subjects (Table 1).

**Table 1:** Bipolar 1 vs. 2 subjects: characteristics

Measure	Measure or Percentage [95%CI]		RR	*p*-value
	BD-1	BD-2	(BD-1/BD-2)	[*t* or χ^2^]
Subjects (n)	657	720	1/1.10	–
*Sex* (%)				0.06 [3.66]
Women	54.3 [50.4–58.2]	59.4 [55.8–63.1]	1/1.09	
Men	45.7 [41.8–49.6]	40.6 [36.9–44.2]	1.13	
*Family history* (%)				
Mood disorders	60.2 [55.8–64.4]	68.4 [64.4–72.2]	1/1.14	0.005 [8.04]
Bipolar disorder	32.6 [28.6–36.8]	30.8 [27.1–34.9]	1.06	0.54 [0.38]
Suicide	9.12 [6.85–11.9]	8.81 [6.69–11.3]	1.04	0.85 [0.04]
*Ages or durations* (years)				
First symptoms	22.4 [21.0–23.8]	27.6 [25.7–29.4]	1/1.23	< 0.0001 [4.24]
Syndromal onset	25.6 [24.8–26.4]	30.3 [29.3–31.4]	1/1.18	< 0.0001 [7.03]
First diagnosis	27.4 [26.0–28.8]	33.7 [31.8–35.6]	1/1.23	< 0.0001 [5.04]
First treatment	27.9 [26.6–29.2]	33.5 [32.0–35.1]	1/1.20	< 0.0001 [5.09]
Symptoms to treatment	5.72 [4.50–6.95]	6.86 [5.67–8.04]	1/1.20	0.19 [1.30]
First suicide attempt	33.2 [31.0–35.4]	36.9 [34.1–39.7]	1/1.11	0.04 [2.07]
Illness duration	17.1 [15.9–18.1]	18.1 [17.1–19.1]	1/1.06	0.17 [1.38]
Current age	43.2 [42.0–44.4]	48.4 [47.2–49.6]	1/1.12	< 0.0001 [6.09]
*Socioeconomic status* (%)				
Educated > high school	19.7 [16.5–23.1]	28.3 [24.8–32.01]	1/1.44	0.0004 [12.4]
Unemployed^a^	16.5 [13.7–19.7]	9.01 [6.96–11.4]	1.83	< 0.0001 [16.7]
Low SES ranking^b^	17.9 [13.7–22.8]	5.32 [3.23–8.19]	3.36	< 0.0001 [18.8]
*Marital history*				
Ever married (%)^c^	38.8 [35.0–42.6]	53.2 [49.4–57.0]	1/1.37	< 0.0001 [28.5]
Ever separated (%)	17.9 [13.9–22.6]	14.7 [11.6–18.4]	1.22	0.24 [1.41]
Children (n/person)	0.80 [0.70–0.90]	1.10 [1.00–1.21]	1/1.38	< 0.0001 [4.11]
*Temperament scores*				
Anxious	1.14 [0.95–1.33]	1.36 [1.21–1.51]	1/1.19	0.07 [1.80]
Cyclothymic	5.21 [4.69–5.73]	6.06 [5.64–6.48]	1/1.16	0.01 [2.49]
Dysthymic	3.15 [2.81–3.49]	3.49 [3.21–3.77]	1/1.11	0.13 [1.51]
Hyperthymic	3.55 [3.20–3.90]	3.65 [2.78–4.52]	1/1.03	0.66 [0.44]
Irritable	1.84 [1.55–2.13]	1.85 [1.59–2.11]	1/1.01	0.98 [0.03]
*Bodyweight*				
BMI (kg/m^2^)	25.0 [24.5–25.5]	24.3 [23.8–247]	1.03	0.04 [2.06]
Obese (%)^d^	21.7 [15.9–28.4]	10.7 [6.35–16.6]	2.03	0.007 [7.37]

**a**. Employed status: employed, students, housewives, and retirees; **b**. less than one salary of approximately 1,000 euros/month for a family of four; **c**. married, divorced, widowed; **d**. BMI≥30

Based on multivariable logistic modeling, BD-2 subjects were significantly and independently more likely to be older at first affective episodes, to have higher SES, and were less obese than BD-1 subjects (Table 6).

### Morbidity comparisons

BD-2 subjects were more likely to have co-occurring medical illnesses and anxiety disorder, but less likely to have ADHD, abuse illicit drugs, and smoked fewer cigarettes/day, but had similar rates of alcohol abuse (Table 2).

**Table 2:** Bipolar 1 vs. 2 disorder: morbidity

Measure	Measure or Percentage [95%CI]		RR	*p*-value
	BD-1	BD-2	(BD-1BD-2)	[*t* or χ^2^]
Subjects (n)	657	720	1/1.10	–
*Co-occurring* (%)				
Medical illness	26.2 [22.9–29.7]	35.9 [32.3–39.5]	1/1.37	< 0.0001 [112]
ADHD	28.7 [23.9–33.8]	21.5 [17.7–25.7]	1.33	0.02 [5.19]
Anxiety disorder	49.5 [42.7–56.4]	60.3 [54.3–66.0]	1/1.22	0.02 [5.72]
*Substance use*				
Drug abuse (%)	20.6 [18.5–24.8]	14.4 [11.9–17.2]	1.43	0.0006 [11.9]
Alcohol abuse (%)	18.7 [14.2–24.0]	13.4 [9.81–17.6]	1.4	0.08 [3.05]
Cigarettes/day	12.3 [10.8–13.7]	9.96 [8.87–11.1]	1.23	0.01 [2.57]
*First episode type* (%)				
Depressive	50.0 [31.9–68.1]	86.5 [71.2–95.5]	1/1.73	< 0.0001 [69.9]
[Hypo]manic	25.0 [11.5–43.4]	10.8 [3.03–25.4]	2.31	< 0.0001 [39.0]
Mixed	9.38 [1.98–25.0]	2.70 [0.07–14.2]	3.47	0.36 [0.85]
Psychotic	15.6 [5.28–32.8]	0.00 [0.00–0.00]	> 15.6	< 0.0001 [136]
Duration (months)	3.16 [2.81–3.52]	5.40 [4.37–6.43]	1/1.71	< 0.0001 [5.17]
*Intake ratings*				
HDRS	14.6 [13.9–15.3]	17.5 [16.9–18.0]	1/1.20	< 0.0001 [6.18]
HARS	7.86 [6.39–9.33]	10.1 [9.00–11.2]	1/1.29	< 0.0001 [5.57]
MADRS	13.6 [11.7–15.5]	17.5 [15.9–19.1]	1/1.29	0.002 [3.15]
MDQ	11.6 [10.8–12.3]	9.56 [8.99–10.1]	1.21	< 0.0001 [4.24]
YMRS	2.40 [1.83–2.97]	1.42 [1.13–1.71]	1.69	0.001 [3.26]
HDRS Improvement (%)	46.2 [29.9–62.4]	63.1 [59.7–66.5]	1/1.37	0.02 [2.36]
*Switch risk* (%)				
Overall	34.2 [22.4–29.2]	49.0 [33.0–40.3]	1/1.43	< 0.0001 [30.8]
Mania	9.13 [7.04–11.6]	0.00 0.00–0.00]	1/1.74	< 0.0001 [68.8]
Hypomania	7.91 [5.97–10.2]	29.6 [26.3–33.1]	1/3.74	< 0.0001 [104]
Mixed	3.96 [98–25.0]	6. 26 [0.07–14.2]	1/1.58	0.002 [10.1]
Psychotic	3.65 [2.35–5.39]	0.14 [0.00–0.77]	26.1	< 0.0001 [23.0]
*Seasonality* (%)				
Fall/winter depression	22.0 [15.5–29.7]	12.4 [7.81–18.3]	1.77	0.02 [5.14]
Spring–summer [hypo]mania	12.8 [7.74–19.4]	1.76 [0.37–5.07]	7.27	0.0001 [14.8]
*Morbidity*				
Episodes/year	2.20 [1.82–2.58]	2.00 [1.23–2.77]	1.1	0.65 [0.45]
Depressions/year	0.84 [0.45–0.82]	1.22 [0.83–1.62]	1/1.45	0.09 [1.70]
[Hypo]manias/year	1.16 [0.80–1.52]	0.89 [0.30–1.49]	1.3	0.46 [0.73]
%-Time ill	39.9 [36.0–43.8]	36.4 [34.0–38.9]	1.1	0.68 [0.42]
%-Time depressed	19.9 [18.0–21.8]	26.4 [24.4–28.4]	1/1.33	< 0.0001 [5.17]
%-Time [Hypo]manic	14.9 [16.4–23.6]	9.53 [8.78–11.3]	248	< 0.0001 [11.1]
Hospitalized (%/year)	8.60 [8.17–9.03]	1.61 [0.74–2.48]	5.34	< 0.0001 [4.27]
DMI course	30.7 [24.3–34.3]	63.3 [58.1–68.0]	1/2.06	< 0.0001 [526]
Predominant depression (%)	58.5 [51.7–65.2]	83.4 [77.4–88.4]	1/1.43	< 0.0001 [30.3]
Rapid or continuous cycling (%)	6.76 [4.87–9.09]	21.4 [18–24.8.2]	1/3.17	< 0.0001 [53.2]
*Suicidal history*				
Ideation only (%)	25.8 [22.4–29.4]	35.2 [31.6–38.9]	1/1.36	< 0.0001 [21.2]
Attempts (%)	20.5 [17.5–23.8]	17.1 [14.4–20.0]	1.2	0.10 [2.71]
Suicide (%)	2.59 [1.51–4.11]	1.53 [0.76–2.72]	1.69	0.16 [1.94]
Violent acts (%)	8.37 [6.37–10.8]	4.86 [3.41–6.70]	1.72	0.008 [6.93]
Lethality (attempts/suicide)	7.94 [5.15–13.3]	11.2 [6.48–22.0]	1/1.41	0.35 [0.89]
Age at first attempt	33.2 [31.1–35.4]	36.6 [34.0–39.2]	1/1.10	0.05 [1.97]

Initial episodes were more likely depressive among BD-2, less likely [hypo]manic, psychotic or with mixed features, and estimated duration of first-lifetime major episodes (usually depressive) was longer in BD-2 subjects. At intake, BD-2 vs. BD-1 subjects had higher standardized ratings for depression and anxiety, but lower scores for [hypo]mania, as expected, and for MDQ ratings for intensity of BD (Table 2). Improvement of initial HDRS_21_ scores with clinical treatment was greater in BD-2 subjects (Table 2), with no differences in changes of YMRS or HARS scores with repeated assessments.

Also of note, BD-2 subjects were more likely to switch mood out of depression during antidepressant treatment, into new hypomania. By definition, BD-2 subjects did not switch into mania, and presented with psychosis rarely and less than with BD-1, as expected (Table 2). Seasonal mood shifts, particularly of [hypo]mania in spring or summer, with somewhat greater risk of fall-winter depression were more likely with BD-1 subjects who also were more likely to follow a rapid- or continuously cycling course (Table 2).

Specific differences in morbidity included higher %-time in depression and predominant depression long-term (≥ 50% of time ill) with BD-2, with less time in [hypo]mania, and much lower risk of psychiatric hospitalization. BD-2 subjects also were more likely to follow the DMI rather than the MDI course-pattern. Risk of suicide and attempts differed little between BD-1 and BD-2 subjects, but violent suicidal acts were nearly twice-more likely with BD-1, whereas BD-2 subjects were more likely to report suicidal ideation without acts. Also, the ratio of suicide attempts/suicide (A/S) was similar with both diagnoses, and lower (greater lethality) than in the general population (Table 2), in which A/S averages 30–40 (Tondo and Baldessarini 2022).

Based on multivariable logistic regression modeling, BD-2 subjects were significantly more likely to follow a DMI course, less likely to be hospitalized, had an older onset-age with fewer years of illness exposure, and to have higher ratings of depression (HDRS) at intake (Table 7).

### HDRS depression item ratings

Based on HDRS_21_ ratings obtained close to intake at the study center, BD-1 and BD-2 subjects differed highly significantly (*p* ≤ 0.01) in total score (20.0% higher with BD-2) and in 16/21 item scores (Table 3). Scores for depression were significantly higher with BD-2 in 13 items, whereas three HDRS item scores were significantly higher among currently depressed BD-1 subjects (Table 3).

**Table 3:** Comparison of HDRS item scores with bipolar-1 vs. bipolar-2 subjects

Number	Item	Mean score [95%CI]		RR	*t*-score	*p*-value
		Bipolar-1	Bipolar-2			
1	Depressed mood	1.98 [1.87–2.10]	2.28 [2.19–2.36]	0.860	4.23	< 0.0001
2	Guilt	0.985 [0.889–1.08]	1.27 [1.19–1.35]	0.774	4.55	< 0.0001
3	Suicidal	0.579 [0.490–0.658]	0.771 [0.696–0.846]	0.754	3.38	0.0008
4	Early insomnia	0.458 [0.386–0.530]	0.660 [0.594–0.726]	0.691	4.01	< 0.0001
5	Middle insomnia	0.435 [0.369–0.501]	0.568 [0.509–0.628]	0.764	2.92	0.004
6	Early awakening	0.432 [0.362–0.503]	0.635 [0.571–0.699]	0.677	4.15	< 0.0001
7	Work and activities	2.16 [2.03–2.29]	2.19 [2.09–2.29]	0.986	0.36	0.72
8	Psychomotor retardation	0.662 [0.587–0.740]	0.490 [0.435–0.546]	1.35	3.67	0.0003
9	Agitation	0.277 [0.221–0.333]	0.414 [0.363–0.465]	0.669	3.45	0.0006
10	Psychic anxiety	1.35 [1.24–1.46]	1.91 [1.83–1.99]	0.702	8.44	< 0.0001
11	Somatic anxiety	0.405 [0.328–0.472]	0.811 [0.730–0.891]	0.493	7.01	< 0.0001
12	Loss of appetite	0.366 [0.312–0.420]	0.475 [0.428–0.522]	0.771	2.98	0.003
13	Fatigue	1.21 [1.13–1.28]	1.31 [1.25–1.37]	0.924	2.04	0.05
14	Anhedonia/genital symptoms	0.884 [0.763–1.01]	0.970 [0.900–1.04]	0.907	1.28	0.18
15	Hypochondriasis	0.163 [0.113–0.212]	0.323 [0.266–0.380]	0.499	3.96	< 0.0001
16	Weight-loss	0.310 [0.256–0.364]	0.409 [0.357–0.461]	0.758	2.55	0.01
17	Insight	0.090 [0.054–0.127]	0.120 [0.088–0.153]	0.750	1.19	0.23
18	Circadian mood variation	0.767 [0.689–0.845]	0.987 [0.919–1.05]	0.777	4.14	< 0.0001
19	Depersonalization/derealization	0.077 [0.044–0.110]	0.056 [0.035–0.076]	1.38	1.14	0.26
20	Paranoid	0.597 [0.506–0.688]	0.364 [0.307–0.421]	1.64	4.49	< 0.0001
21	Obsessive–compulsive	0.071 [0.039–0.102]	0.102 [0.073–0.131]	0.689	1.43	0.15
1–21	Total score	14.5 [13.7–15.2]	17.4 [16.8–17.9]	0.833	6.42	< 0.0001

In multivariable logistic regression modeling, factor which remained significantly and independently greater with BD-2 than BD-1 ranked: more psychic anxiety, less psychomotor retardation, less paranoia, more somatic anxiety, more suicidal, more early awakening, and more hypochondriasis (Appendix: Table 8).

### Treatment comparisons

Long-term treatment with lithium was less prescribed to BD-2 subjects, and average daily doses of lithium salts were lower with BD-2, although mean (individualized) daily minimum serum concentrations of lithium were similar with both diagnostic groups, as were uses of other psychotropic agents with lithium (Table 4). Among other psychotropic drugs, antidepressants and benzodiazepines were more used by BD-2 subjects, whereas antipsychotics were more often given to BD-1 subjects (Table 4). Use of mood-stabilizing anticonvulsants and participation in psychotherapy did not differ by diagnosis (Table 4).

**Table 4:** Bipolar 1 vs. 2 subjects: treatment

Measure	Mean or percentage [95%CI]		RR	*p*-value
	BD-1	BD-2	(BD-1/BD-2)	*[t* or χ^2^]
Subjects (n)	657	720	1/1.10	–
*Lithium use*				
Treated (%)	65.0 [61.2–68.6]	49.6 [45.9–53.3]	1.27	< 0.0001 [33.3]
Dose (mg/day)	828 [796–860]	609 [577–641]	1.36	< 0.0001 [9.46]
Mean level (mEq/L)	0.61 [0.58–0.65]	0.56 [0.51–0.61]	1.09	0.11 [1.62]
Lithium + other drugs (%)	20.6 [16.4–25.3]	18.0 [13.2–23.6]	1.14	0.44 [0.59]
*Other treatments* (%)				
Antidepressants	41.7 [37.3–46.2]	71.0 [67.0–74.8]	1/1.70	< 0.0001 [90.2]
Anticonvulsants	39.1 [35.4–42.9]	34.3 [30.9–37.8]	1.14	0.06 [3.62]
Antipsychotics	19.9 [16.9–23.0]	8.20 [6.33–10.4]	2.43	< 0.0001 [40.8]
Benzodiazepines	8.76 [6.75–11.1]	15.7 [13.2–18.5]	1/1.79	< 0.0001 [16.0]
Psychotherapy	40.1 [34.4–50.8]	45.4 [40.4–50.8]	1/1.13	0.17 [1.88]

In multivariable logistic regression modeling, significant and independent differences favoring BD-2 subjects included more antidepressant treatment, and less treatment with antipsychotics or lithium (Appendix: Table 9).

### Summary comparisons

Factors found to be highly significantly (*p* ≤ 0.01 to limit effects of multiple comparisons) different between BD-2 and BD-1 study subjects (Tables 1, 2, 3, 4) are summarized in Table 5.

**Table 5:** Highly significant differences between BD-2 and BD-1 subjects

More with BD-2	More with BD-1
Familial mood disorders	Obesity
Older at: first symptoms, syndrome, diagnosis, treatment	Drug and alcohol abuse; cigarettes/day
Educated > high school	First episode [hypo]manic
Employed	First episode psychotic
Ever married	Higher intake scores: YMRS, MDQ
Children	Spring–summer seasonal [hypo]mania
Higher SES rating	%-Time [hypo]manic
Cyclothymic temperament	Psychiatric hospitalizations/year
Co-occurring general medical and anxiety disorders	Violent suicidal acts
First lifetime episode: depressive and longer	Use of lithium and mg/day doses
Intake depression (HDRS, MADRS) and anxiety (HARS)	HDRS items: psychomotor retardation, paranoia
HDRS items: depressed mood, guilt, suicidal, insomnia, agitation, psychic and somatic anxiety, hypochondriasis, weight-loss, circadian mood-fluctuation, total score	
%-Time depressed	
Predominant depression	
DMI course pattern	
Rapid or continuous cycling	
Prescribed antidepressants and benzodiazepines	

All measures differ at *p* ≤ 0.01

## Discussion

In this large cohort of bipolar disorder patients from a specialized outpatient clinic, we found significant differences between BD-2 vs. BD-1. First, there was a nearly 10% excess of BD-2 diagnoses, compared to an average of 1.48-fold [1.45–1.50] excess of BD-1 over BD-2 cases among clinical samples in recent reports (Bega et al. 2012; Dell'Osso et al. 2017; Karanti et al. 2020; Guzman-Parra et al. 2021; Clemente et al. 2015), and international epidemiological data indicating lifetime prevalence of BD-1/BD-2 of 0.6%/0.4% (ratio = 1.50) in the general population (Merikangas et al. 2011). Variations in prevalence may depend whether subjects are or were ever hospitalized (biasing toward BD-1) or have been outpatients. 

Among the present subjects, women and men did not differ in risk for either BD-1 or BD-2, although the proportion of women among all subjects was higher, as often is found with clinical samples of mood-disorder patients (Viguera et al. 2001). We found a higher rate of family history for mood disorders among BD-2 subjects in general, but not for BD specifically nor for suicidal behavior (Table 1), possibly indicating misclassifications in relatives of BD-2 subjects. Among ratings of affective temperaments, only cyclothymic temperament scores differed between the diagnoses and favored BD-2.

Not surprisingly, diagnosis and initial treatment occurred later among BD-2 than BD-1 subjects, although intervals between initial symptoms and eventual diagnosis and start of treatment were similar in both groups because of later onset and later age at intake reflecting less clinically urgent early morbidity with BD-2 vs. BD-1 (Table 1). Of note, the time from illness onset to first treatment (2.3 years in BD-1 and 3.2 in BD-2) is similar to that of 2.6 years reported in another large Italian cohort of BD patients overall (Dell'Osso et al. 2017). These relatively brief latencies probably reflect improved early recognition of BD.

Consistent with other observations (Buoli et al. 2021), the first lifetime episode was more likely depressive than [hypo]manic or mixed, and was of longer duration in BD-2 vs. BD-1 subjects (Table 2). The first suicide attempt occurred about 7 years after illness-onset in both groups, but three years later with BD-2 vs. BD-1. Depression was the predominant polarity in BD-2 subjects who also were more likely to follow a long-term DMI course (Table 2), as we had found previously (Kukopulos et al. 1980; Baldessarini et al. 2012). In addition, BD-2 subjects were more prone to switch from depression to elevated mood in association with antidepressant treatment, consistent with their tendency to follow a DMI course and lesser likelihood of receiving mood-stabilizing agents. BD-2 subjects were less subject to seasonal variations than BD-1 subjects, unlike findings from a smaller recent study (Yeom et al. 2021). The greater likelihood of having a cyclothymic temperament and a rapid-cycling course along with less seasonal changes may indicate that mood oscillations are more chaotic in BD-2 vs. BD-1 subjects. Psychometric scores for depression and anxiety were higher at intake among BD-2 patients, whereas ratings of lifetime (MDQ) and current [hypo]manic symptoms (YMRS) were higher with BD-1, all as expected (Table 2).

Noteworthy findings support the conclusion that education, employment, and socioeconomic status were more favorable among BD-2 subjects, and they were more likely to be married and to have children (Table 1). These differences suggest that BD-2 patients face less adverse functional impact than with BD-1. The lower risk of psychiatric hospitalization among BD-2 subjects (Table 2) was expected since BD patients require hospitalization predominantly for manic or psychotic episodes or suicidal risk. However, both syndrome types were associated with similar illness intensity as measured by episodes/year and by the overall proportion of time ill. BD-2 subjects spent more time in depression overall, even though the duration of depressive episodes was similar with BD-2 and BD-1 subjects, averaging 5.28 [4.99–5.58] months for depression and 3.39 [3.11–3.66] months for [hypo]mania (not shown), consistent with a higher number of depressive episodes with BD-2 (Table 2). Risks for suicides and attempts were similar in both diagnoses, although BD-2 subjects had more suicidal ideation, and BD-1 was more associated with violent suicidal acts (Table 2).

General health also may differ between the BD types. Obesity was much more likely with BD-1 (Table 1), as we reported recently (Miola et al. 2021). Weight-gain may arise from complex treatment regimens typically encountered among BD-1 patients, often including appetite-increasing medicines (Tondo and Baldessarini 2022). In contrast, BD-2 subjects were more likely to have co-occurring general medical and anxiety disorders, but less likely to experience have ADHD or to experience drug abuse (Table 2), whereas risk of abuse of alcohol was similar with both diagnoses.

As expected and confirming a recent report (Shinozaki et al. 2022), a high proportion of about 85% of BD-1 subjects received long-term treatment with lithium alone or in combination with other mood-altering medicines compared to 68% of BD-2 patients. The proportion of patients treated with lithium is expectedly high as the study center specializes in such treatment. BD-1 subjects received higher doses of lithium in order to provide similar plasma lithium concentrations as in BD-2 subjects. The difference in dosing may reflect the younger age of BD-1 subjects. Also unsurprisingly, antipsychotics were prescribed more frequently for BD-1 than for BD-2 patients, whereas antidepressants and benzodiazepines were more prevalent with BD-2 (Table 4).

The present findings indicate that the major DSM-5 BD subtypes differed in many demographic, socioeconomic, and other clinical measures, either cross-sectional or course-related, and that they were treated differently. Although BD-2 subjects did relatively well functionally, based on education, employment, socioeconomic indicators as well as by marriage and parenting, probably in association with later illness onset, which was intermediate between BD-1 and MDD. Instead, the two BD syndromes differed in their psychopathological and functional patterns, with depression as the dominant component in BD-2 with less prominent elevation of mood and behavior in hypomania, in contrast to characteristic mania, sometimes with psychotic features, as well as depression in BD-1. With all of these characteristics considered, it does not seem accurate to consider BD-2 as a less severe form of BD.

Differences between BD-2 and BD-1 highlighted in this study represent more than an academic exercise in nosology or psychopathology, and are aimed at supporting the search for optimal treatments for both syndromes—particularly for the depressive polarity which is still considered a major unmet need (Baldessarini 2013). Notable differences between the two syndromes include severe mood elevations in BD-1, with less favorable functional outcomes. With BD-2, the later onset, less elevated mood, and rarity of psychosis are compatible with superior functioning. On the contrary, depressive polarity was more prominent with BD-2 than with BD-1. These differences alone would justify the two-syndrome concept, just as depressive episodes without episodes of [hypo]mania are required for a DSM-5 diagnosis of MDD. A largely neglected feature in BD is the course sequence. Specifically, in BD-1, depression often follows a manic episode (MDI course, found in 70% of the present BD-1 cases) and it may be treated by simply decreasing or stopping antimanic or antipsychotic agents or perhaps adding an antidepressant temporarily as needed. BD-2, instead, usually follows a DMI course (63% of the present BD-2 cases), which requires particular care in the use of antidepressants since they can induce a [hypo]manic episode or accelerate the illness course up to rapid-cycling (Kukopulos et al. 1980). All these differing factors and their prognostic implications are important to discuss with BD patients or, sometimes even more importantly, with their relatives.

Based on the present findings, differentiation of two major BD syndromes seems to be justified. Judd and colleagues (Judd et al. 2003), although recognizing some dimensional characteristics in BD subtypes found, in a relatively small cohort of well-evaluated BD patients, sufficient distinctive characteristics to support the separation of the two diagnostic subtypes, confirmed by a much larger, cross-sectional study by Swedish investigators, based on DSM-IV diagnostic criteria and a national register database (Karanti et al. 2020). Differentiation of the two nosologic entities within BD is in line with current interest in developing more precise and individualized treatments throughout medicine, including in psychiatry (Alda 2013). For now, even more research is needed to address the differentiation of BD-2 from MDD patients and to demonstrate their optimal short- and long-term treatment (Swartz and Suppes 2019; Vieta and Suppes 2008).

We suggest that the subtypes of BD disorders should use Arabic numerals 1 and 2 instead of Roman ordinal numbers I (first) and II (second) to correspond with spoken language and in accord with the shift of DSM editions to Arabic numerals. Future revisions of DSM should include codes for BD-2 indicating the type of current or last episode, illness severity, and remission state as are now provided for BD-1 as does the ICD-11 (codes 6A61.x) which includes also the presence of psychotic symptoms during the depressive polarity in BD-2 (ICD-11,2022). Differences in morbidity and psychometric scores may indicate that BD-2 patients differ from BD-1, not as a less severe manifestation of a single disorder, but as a distinct syndrome supported by many demographic, descriptive, assessment, and treatment differences (Tables 1, 2, 3, 4, 5) (Karanti et al. 2020), as well as by new findings from a large genome-wide association study finding a higher genetic correlation of BD-1 with schizophrenia, but a stronger relationship of BD-2 to MDD (Mullins et al. 2021).

We agree that a dimensional model of BD (Malhi et al. 2020; Gitlin and Malhi 2020) reflects more closely the variability of a complex illness, as was recognized early by Klerman (1981), but use of a categorical model is more immediate for epidemiological and genomic studies, routine clinical use, scientific communication, and helpful for systematic classifications.

### Limitations

Some differences found between cases of BD-2 and BD-1 reflect accepted definitions of the syndromes, and lack of mania or psychosis with BD-2 virtually assures higher functioning. Retrospectively ascertained differences are subject to recall biases, but are likely to arise similarly with both syndromes. However, as in all studies including BD-2 patients and despite efforts to identify retrospective hypomanic episodes, some mild hypomanias may have passed undetected, among patients eventually diagnosed with MDD. Treatments were assigned clinically and may represent standard local practice patterns.

### Conclusions

BD-2 cases were characterized by more prominent and longer depressions with some hypomania and mixed-features but not mania and rarely psychosis. They also had higher socioeconomic and functional status but also high levels of long-term morbidity and suicidal risk. Accordingly, BD-2 is dissimilar to, but not necessarily less severe than BD-1. The several prominent dissimilarities between BD-1 and BD-2 support the hypothesis that they represent distinct syndromes in need of individualized treatments.

## Data Availability

The datasets generated or analyzed during the current study are not publicly available due to confidentiality agreement with study participants but are available from the corresponding author on reasonable request, with confidentiality restrictions.

## Appendix

See Tables 6, 7, 8, 9.

**Table 6:** Logistic multivariable model for characteristics associated with BD-2 > BD-1

Factor	OR [95% CI]	χ^2^	*p*-value
Later onset age	1.04 [1.02–1.06]	15.1	0.0001
Higher SES	4.25 [1.90–9.54]	12.3	0.0004
Less likely obese	2.60 [1.27–5.33]	6.83	0.009

**Table 7:** Logistic multivariable model for morbidity associated with BD-2 > BD-1

Factor	OR [95% CI]	χ^2^	*p*-value
DMI course	3.64 [2.25–5.86]	33.5	< 0.0001
Fewer hospitalizations/year	38.0 [6.78–213]	21.1	< 0.0001
Older onset-age	1.03 [1.02–1.05]	9.73	0.002
Fewer total years ill	1.02 [1.01–1.04]	5.31	0.02
Intake HDRS score	1.04 [1.01–1.07]	4.91	0.03

**Table 8:** Logistic multivariable model for HDRS item scores associated with BD-2 > BD-1

Factor	OR [95% CI]	χ^2^	*p*-value
Psychic anxiety	1.43 [1.27–1.62]	33.2	< 0.0001
Less retardation	1.58 [1.32–1.87]	25.1	< 0.0001
Less paranoia	1.41 [1.21–1.64]	19.6	< 0.0001
Somatic anxiety	1.40 [1.20–1.62]	19.0	< 0.0001
Suicidal	1.22 [1.05–1.42]	6.41	0.01
Early wakening	1.18 [1.01–1.39]	4.15	0.04
Hypochondriasis	1.23 [1.00–1.53]	3.70	0.05

**Table 9:** Multivariable logistic regression model for treatment: associated with BD-2 > BD-1

Factor	OR [95% CI]	χ^2^	*p*-value
Antidepressant use	6.77 [5.22–8.79]	207	< 0.0001
No antipsychotics	5.91 [4.10–8.53]	90.3	< 0.0001
No lithium	2.01 [1.57–2.57]	31.2	< 0.0001
